# Clinical Efficacy of Neoadjuvant Camrelizumab Plus Chemotherapy for Triple‐Negative Breast Cancer and Tumor Immune Microenvironment Analysis: A Prospective‐Retrospective Cohort Study

**DOI:** 10.1002/mco2.70924

**Published:** 2026-08-17

**Authors:** Yongsheng Wang, Pengfei Qiu, Yuechen Wang, Xingchen Meng, Jiaxin Zhang, Haiqiang Liu, Zhiqiang Shi, Zhaopeng Zhang, Xiao Sun, Chunjian Wang, Xiangjing Meng, Chunhui Zheng

**Affiliations:** ^1^ Breast Cancer Center Shandong Cancer Hospital and Institute Shandong First Medical University and Shandong Academy of Medical Sciences Jinan China; ^2^ Breast Disease Center Weifang People's Hospital Shandong Second Medical University Weifang China; ^3^ Department of Anaesthesiology Weifang People's Hospital Shandong Second Medical University Weifang China; ^4^ Toxicology Research Center Shandong Academy of Occupational Health and Occupational Medicine Shandong First Medical University & Shandong Academy of Medical Sciences Jinan China

**Keywords:** camrelizumab, immunotherapy, neoadjuvant chemotherapy, triple‐negative breast cancer, tumor immune microenvironment

## Abstract

The impact of immune checkpoint inhibitors combined with neoadjuvant chemotherapy (NAC) on surgical outcomes and immune remodeling remains underexplored. To investigate the efficacy and tumor immune microenvironment (TIME) changes of camrelizumab plus NAC for triple‐negative breast cancer (TNBC), we collected TNBC patients receiving neoadjuvant immunochemotherapy (NIC) or NAC from clinical trials and routine practice, and set a series of endpoints, including total pathological complete response (pCR), breast pCR and TIME characteristics. After propensity score matching, the NIC cohort showed significantly higher rates of total pCR, breast pCR, and axillary pCR, and a lower rate of axillary lymph node dissection than the NAC cohort (all *p* < 0.05). Within the NIC group, patients achieving total pCR (*p* = 0.012) or breast pCR (*p* = 0.0078) had superior disease‐free survival. Analysis of TIME characteristics indicated that the 7 TIME signatures, including tumor inflammation signature, antigen processing machinery and T cell‐related features, could predict pCR response to NIC. The finding suggested that in TNBC, NIC enhances pCR achievement and reduces axillary dissection risk. Elevated TIME profiles of the 7 signatures may predict favorable pCR response to NIC.

## Graphical abstract

**Cohort Study Design**:Between January 2019 and July 2023, 276 stage II‐III TNBC patients were enrolled in this study, including 56 in the NIC cohort and 220 in the NAC cohort. Due to 17 cases of ineligible criteria, 5 with withdrawal of informed consent, 198 patients in the NAC cohort completed NAC and underwent surgical resection. In the NIC cohort, 54 patients completed NIC and received surgical resection after excluding 2 of discontinuation.

**Prognositic Analysis**:After propensity score matching, the NIC cohort showed significantly higher rates of total pCR, breast pCR, and axillary pCR, and a lower rate of axillary lymph node dissection than the NAC cohort (all *p* < 0.05). Within the NIC group, patients achieving total pCR (*p* = 0.012) or breast pCR (*p* = 0.0078) had superior disease‐free survival.

**TIME Characteristics Analysis**:To further explore the correlation between TIME characteristics and DFS, we performed a correlation analysis between the 7 differential signatures specific to pCR patients in the NIC group and DFS. By dividing the patients into high‐ and low‐score groups according to the median score of the 7 signatures, we found that the high‐score group seemed to have better DFS than the low‐score group, but no statistical difference was presented.

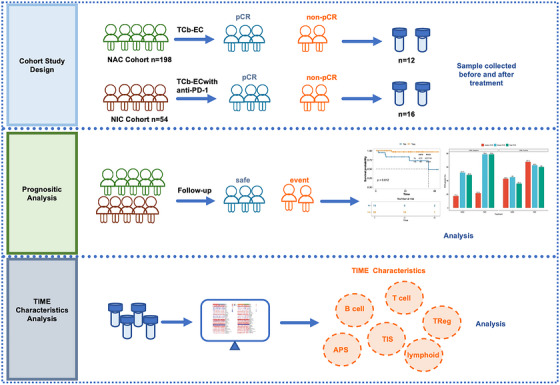

## 1 Introduction

Triple‐negative breast cancer (TNBC), accounting for approximately 15%–20% of all breast cancer cases [1], confers a higher risk of early recurrence, distant metastasis, and inferior overall survival (OS) relative to other breast cancer subtypes. The lack of targeted therapies renders chemotherapy the primary treatment modality, with neoadjuvant chemotherapy (NAC) playing a pivotal role in diminishing tumor burden and optimizing surgical outcomes [2]. By contrast to those with residual diseases, breast cancer patients achieving complete pathological response (pCR) following NAC exhibit better survival outcomes, yet only 30%–40% of patients can attain pCR, highlighting the need for more effective therapeutic strategies [3, 4].

Currently, breakthroughs in cancer immunotherapy have opened new avenues for TNBC treatment. Combination of immune checkpoint inhibitors (ICIs) with NAC has shown promise in increasing pCR rates in early‐stage TNBC. The CamRelief trial demonstrated that the addition of camrelizumab to NAC significantly increased pCR rates and event‐free survival in patients with high‐risk TNBC [5]. However, the impact of this combination on surgical outcomes, such as the likelihood of breast‐conserving surgery (BCS) and the potential to avoid axillary lymph node dissection (ALND), remains underexplored.

The tumor immune microenvironment (TIME), a critical determinant of response to immunotherapy, is strongly associated with tumorigenesis, recurrence, and metastasis [6]. Breast cancer has long been thought to be a “cold” tumor because of its limited T‐cell infiltration and low tumor mutational burden. However, TNBC is characterized by abundant tumor‐infiltrating lymphocytes, which creates a favorable TIME and confers responsiveness to ICIs [7]. The evidence has indicated that the TIME status may serve as a biomarker to predict the response to antitumor therapy, and the components in tumor microenvironment can impact the tumorigenesis and the response to NAC [8, 9]. To date, the dynamic remodeling of the TIME induced by NAC with or without immunotherapy and its correlation with clinical outcomes are not fully understood.

In this study, we investigated the clinical efficacy of camrelizumab plus NAC for TNBC, focusing on its impacts on surgical and short‐term outcomes. In addition, the changes in the TIME before and after treatment were also explored.

## 2 Results

### 2.1 Patient Characteristics

Between January 2019 and July 2023, 276 stage II–III TNBC patients were enrolled in this study, including 56 in the NIC cohort and 220 in the NAC cohort. Due to 17 cases of ineligible criteria, 5 with withdrawal of informed consent, 198 patients in the NAC cohort completed NAC and underwent surgical resection. In the NIC cohort, 54 patients completed NIC and received surgical resection after excluding 2 of discontinuation. There were 252 patients included in the oncological outcome analysis.

**FIGURE 1:**
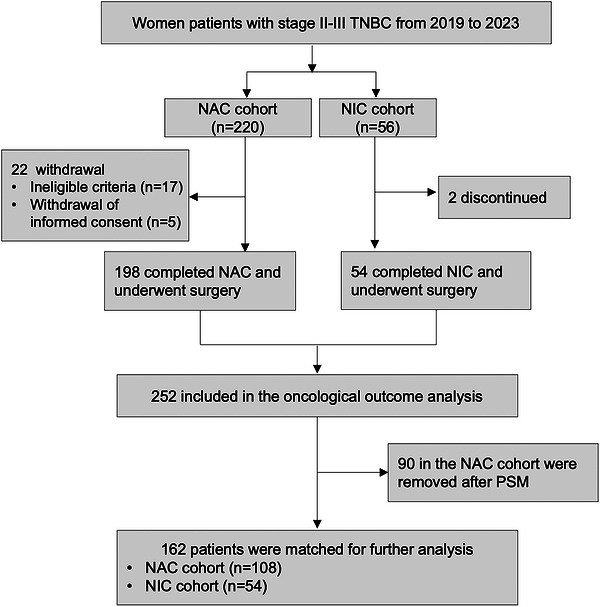
The profile of study cohorts.

To balance the potential bias, propensity score matching (PSM) was conducted based on the patient age, tumor stage T, stage N, and Ki67. When 90 cases in the NAC cohort were removed after PSM, a total of 162 patients were matched for further analysis, including 108 in the NAC cohort and 54 in the NIC cohort (Figure 1). The baseline characteristics of both cohorts are presented in Table 1 before and after PSM. It could be observed that there were no significant differences in the clinicopathologic features between the NAC and NIC cohorts whether before or after PSM (all *p* > 0.05; Table 1).

**TABLE 1:** Baseline characteristics of the study cohorts.

Characteristics	Before PSM		*p*	After PSM		*p*
	NAC (*n* = 198)	NIC (*n* = 54)		NAC (*n* = 108)	NIC (*n* = 54)	
**Age, years**			0.055			1.000
< 60	164 (82.8)	51 (94.4)		102 (94.4)	51 (94.4)	
≥ 60	34 (17.2)	3 (5.6)		6 (5.6)	3 (5.6)	
**Menopausal status**			1.000			0.78
Post	94 (47.5)	25 (46.3)		46 (42.6)	25 (46.3)	
Pre	104 (52.5)	29 (53.7)		62 (57.4)	29 (53.7)	
**Clinical stage T**			0.864			0.999
0	2 (1.0)	0 (0.0)		0 (0.0)	0 (0.0)	
1	32 (16.2)	8 (14.8)		17 (15.7)	8 (14.8)	
2	115 (58.1)	33 (61.1)		65 (60.2)	33 (61.1)	
3	28 (14.1)	9 (16.7)		18 (16.7)	9 (16.7)	
4	21 (10.6)	4 (7.4)		8 (7.4)	4 (7.4)	
**LN status**			0.261			0.629
0	29 (14.6)	14 (25.9)		19 (17.6)	14 (25.9)	
1	92 (46.5)	21 (38.9)		43 (39.8)	21 (38.9)	
2	49 (24.7)	13 (24.1)		32 (29.6)	13 (24.1)	
3	28 (14.1)	6 (11.1)		14 (13.0)	6 (11.1)	
**Ki‐67**			0.211			1.000
<30%	30 (15.2)	4 (7.4)		8 (7.4)	4 (7.4)	
≥30%	168 (84.8)	50 (92.6)		100 (92.6)	50 (92.6)	

*Note*: Data are *n* (%) unless otherwise indicated.

Abbreviations: LN, lymph nodes; NAC, neoadjuvant chemotherapy; NIC, neoadjuvant immunochemotherapy; PSM, propensity score matching.

### 2.2 Surgical and Pathological Outcomes

Surgical and pathological outcomes of the patients undergoing surgery are summarized in Table 2. Patients undergoing NIC exhibited no significant difference in BCS rates compared with those treated with NAC before PSM (79.6% vs. 82.3%, *p* = 0.798) and after PSM (79.6% vs. 84.3%, *p* = 0.607). The proportion of patients not receiving ALND in the NIC cohort was significantly lower than those in the NAC cohort whether before PSM (61.1% vs. 87.4%, *p* < 0.001) or after PSM (61.1% vs. 87.0%, *p* < 0.001). The total pathological complete response (pCR), breast pCR, and axillary pCR rates of patients treated with NIC before PSM were 64.8%, 66.7%, and 55.6%, significantly higher than 37.4% (*p* = 0.001), 46.0% (*p* = 0.011), and 38.9% (*p* = 0.003) of the patients treated with NAC. Notably, the patients treated with NIC still showed higher total pCR, breast pCR, and axillary pCR rates than those treated with NAC after PSM (64.8% vs. 37.0%, *p* = 0.001; 66.7% vs. 47.2%, *p* = 0.030; 55.6% vs. 34.3%, *p* = 0.007; Table 2).

**TABLE 2:** Surgical and pathological outcomes in the patients receiving surgery.

Characteristics	Before PSM		*p*	After PSM		
	NAC (*n* = 198)	NIC (*n* = 54)		NAC (*n* = 108)	NIC (*n* = 54)	*p*
**Surgical types**			0.798			0.607
BCS	163 (82.3)	43 (79.6)		91 (84.3)	43 (79.6)	
Mastectomy	35 (17.7)	11 (20.4)		17 (15.7)	11 (20.4)	
**ALND**			< 0.001			< 0.001
No	173 (87.4)	33 (61.1)		94 (87.0)	33 (61.1)	
Yes	25 (12.6)	21 (38.9)		14 (13.0)	21 (38.9)	
**Total pCR**			0.001			0.001
No	124 (62.6)	19 (35.2)		68 (63.0)	19 (35.2)	
Yes	74 (37.4)	35 (64.8)		40 (37.0)	35 (64.8)	
**Breast pCR**			0.011			0.030
No	107 (54.0)	18 (33.3)		57 (52.8)	18 (33.3)	
Yes	91 (46.0)	36 (66.7)		51 (47.2)	36 (66.7)	
**Miller/Payne grading**			0.063			0.066
1	5 (2.5)	0 (0.0)		4 (3.7)	0 (0.0)	
2	36 (18.2)	5 (9.3)		19 (17.6)	5 (9.3)	
3	35 (17.7)	9 (16.7)		17 (15.7)	9 (16.7)	
4	33 (16.7)	4 (7.4)		18 (16.7)	4 (7.4)	
5	88 (44.4)	36 (66.7)		50 (46.3)	36 (66.7)	
**Axillary pCR**			0.003			0.007
No	95 (48.0)	12 (22.2)		51 (47.2)	12 (22.2)	
None	26 (13.1)	12 (22.2)		20 (18.5)	12 (22.2)	
Yes	77 (38.9)	30 (55.6)		37 (34.3)	30 (55.6)	

*Note*: Data are *n* (%) unless otherwise suggested.

Abbreviations: ALND, axillary lymph node dissection; BCS, breast‐conserving surgery; NAC, neoadjuvant chemotherapy; NIC, neoadjuvant immunochemotherapy; pCR, pathological complete response; PSM, propensity score matching.

**FIGURE 2:**
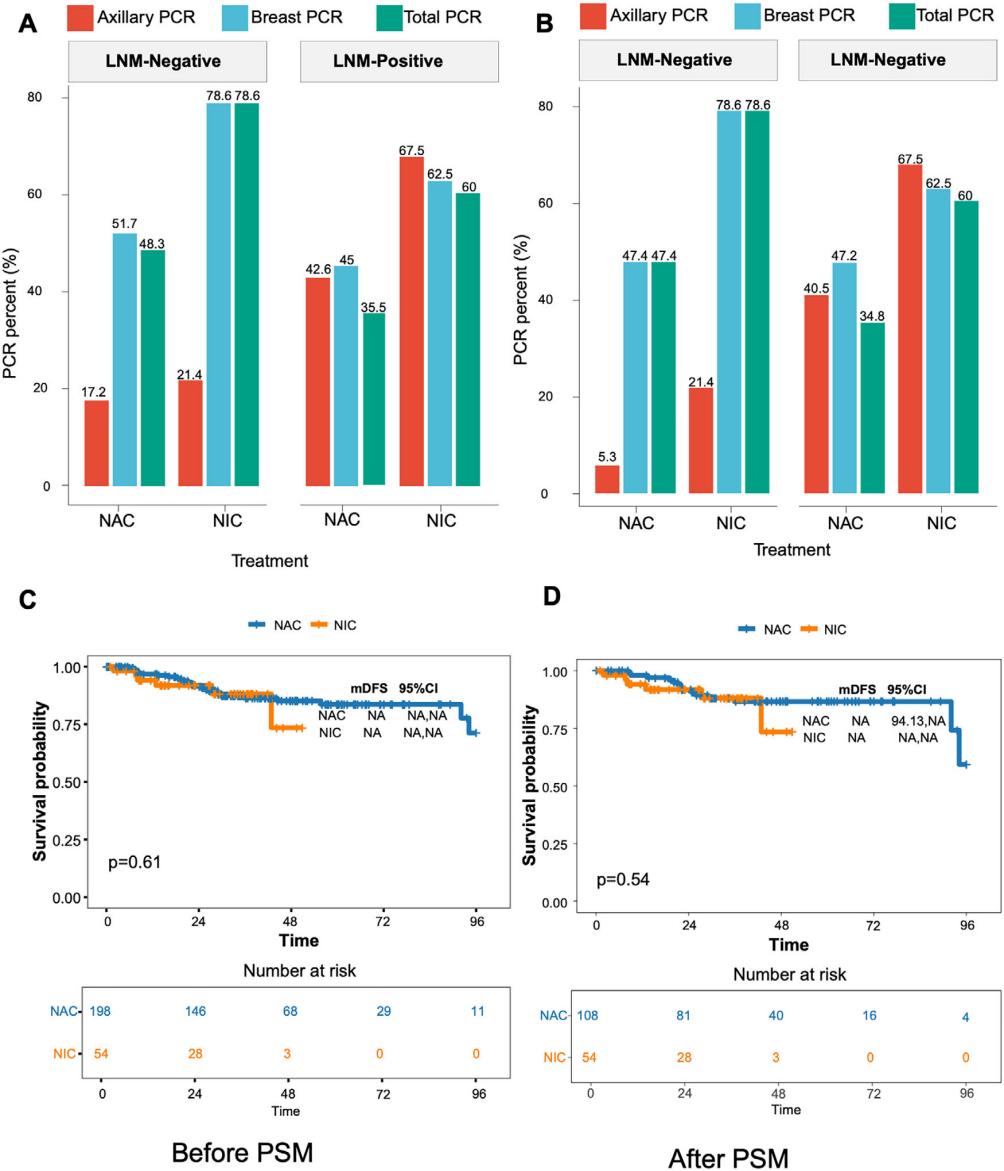
Pathological response rates of TNBC patients treated with NIC versus NAC based on the status of lymph nodes (A). The red, blue and green bars indicate axillary, breast, and total pCR rates, respectively. DFS analysis of TNBC patients treated with NIC versus NAC before and after PSM (B).

Before PSM, the NIC cohort had 40 node‐positive and 14 node‐negative patients, while 169 node‐positive and 29 node‐negative patients in the NAC cohort. After PSM, the NIC cohort retained the same nodal distribution, and the matched NAC cohort included 89 node‐positive and 19 node‐negative patients. Subgroup analysis further indicated that the axillary pCR, breast pCR, and total pCR rates of node‐positive patients receiving NIC were all significantly higher than those receiving NAC before PSM, and these significant differences still existed after PSM (all *p* < 0.05; Figure 2A).

### 2.3 Disease‐Free Survival

**FIGURE 3:**
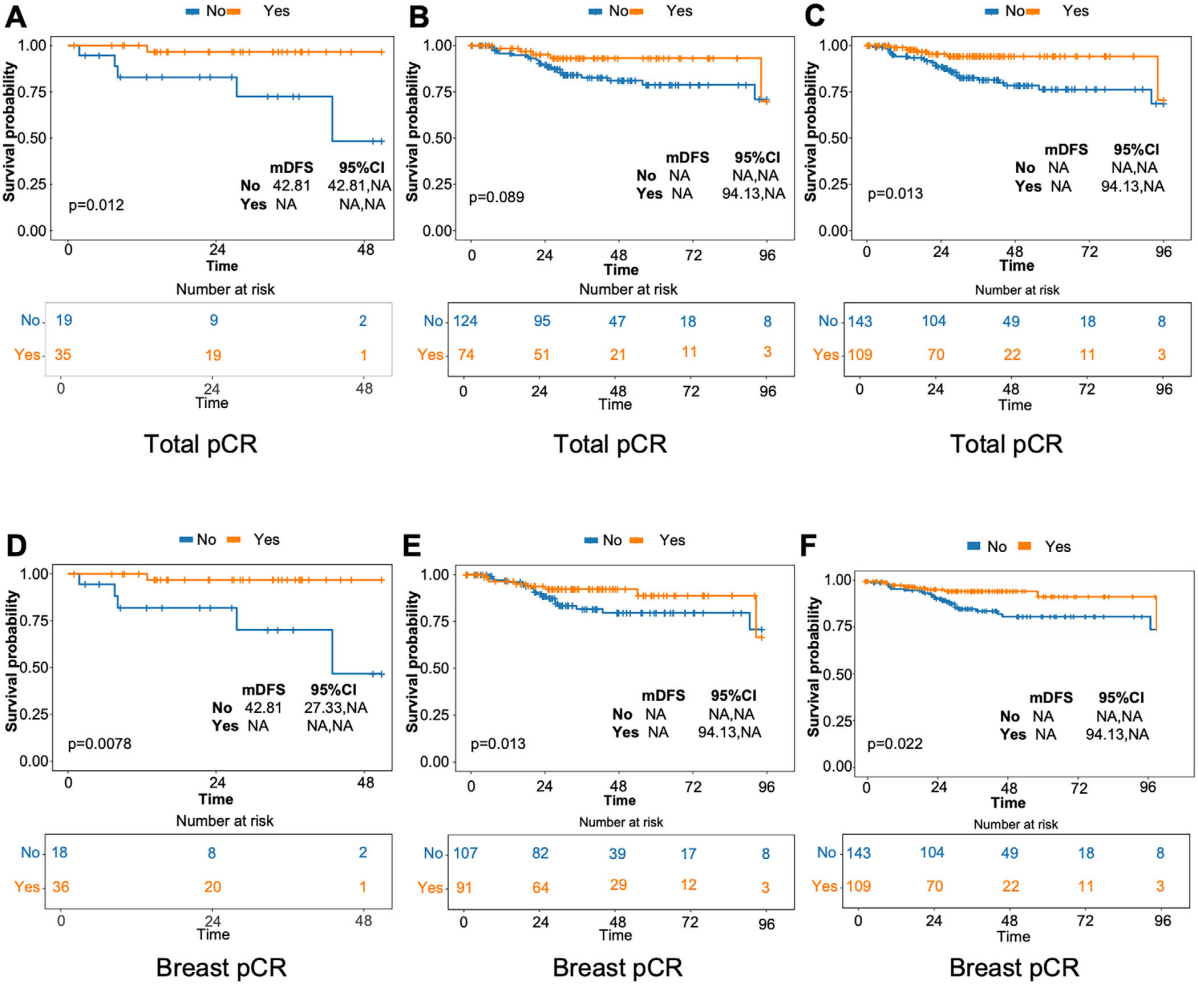
DFS analysis of TNBC patients stratified by achievement of total pCR in NIC cohort (A), NAC cohort (B) and overall combined cohort (C); and breast pCR shown for the NIC cohort (D), NAC cohort (E) and overall combined cocort (F).

The database was locked on March 5, 2025. The median follow‐up period was 35.5 months (33–41 months). By comparison to the disease‐free survival (DFS) between NIC and NAC groups, no significant differences were presented whether before PSM (*p* = 0.63, median not reached) or after PSM (*p* = 0.54, median not reached; Figure 2B), which might be associated with the limited sample size and short‐term follow‐up duration. Subsequently, the DFS of patients from both cohorts was further analyzed according to the stratification of the presence or absence of achieving total pCR and breast pCR. It could be observed that the patients with total pCR (*p* = 0.012, median not reached) and breast pCR (*p* = 0.0078, median not reached) in the NIC cohort had better DFS than those with residual tumors, but no significant differences in the NAC cohort (*p* = 0.089, 0.17, respectively; Figure 3A,B). In addition, regardless of treatment regimens, the patients achieving total pCR and breast pCR in the overall cohort had significantly prolonged DFS than those not achieving total pCR (*p* = 0.013, median not reached) or breast pCR (*p* = 0.022, median not reached) (Figure 3C).

### 2.4 Unique TIME Characteristics of pCR Patients Treated by NIC

**FIGURE 4:**
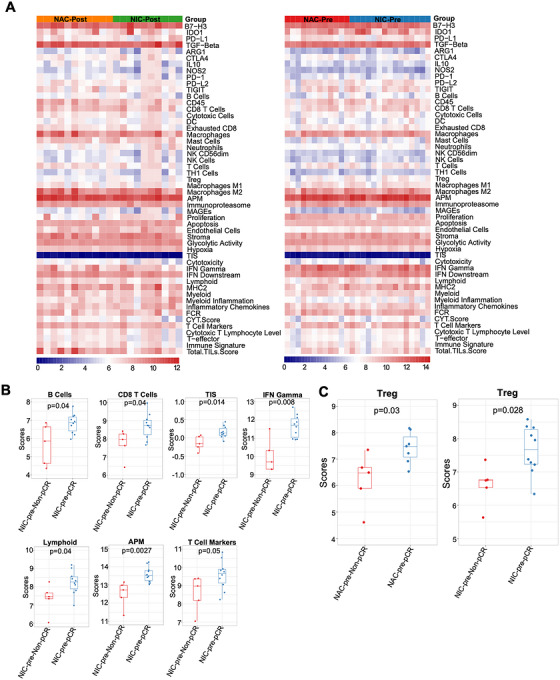
The heatmaps of the pre‐ and post‐treatment TIME between the NIC and NAC cohorts (A), 7 differential signatures identified between pCR and non‐pCR patients form the NIC cohort before treatment (B), and comparison of the pre‐treatment Treg cell infiltration in pCR patients from the NIC and NAC cohorts with non‐pCR patients (C).

No significant difference was shown in the gene expression signature between the pre‐treatment NIC and NAC samples, indicating that the pre‐treatment TIME status between the two groups was basically consistent. By comparing the post‐treatment TIME status between the two groups, we observed one significant signature (Figure 4A).

To explore the TIME characteristics of pCR patients, we compared the pre‐treatment differences in TIME characteristics between pCR and non‐pCR patients in the NIC and NAC groups. Totally 24 differentially expressed genes (DEGs) and 7 differential signatures were identified between pCR and non‐pCR patients in the NIC group before treatment. Among the 24 DEGs, there were 9 genes with higher expression and 15 genes with lower expression in pCR patients compared with non‐pCR patients. Notably, 7 differential signatures, including B cells, CD8^+^ T cells, tumor inflammation signature (TIS), interferon (IFN) gamma, lymphoid, antigen processing machinery (APM), and T cell markers, were all significantly higher in pCR patients (Figure 4B). However, in the NAC group, there were 12 DEGs and 1 differential signature (Treg cells). Of the 12 DEGs, there were 7 genes with higher expression and 5 genes with lower expression in pCR patients compared with non‐pCR patients, and the signature of Treg cells was also significantly higher in pCR patients. Likewise, the proportion of Treg cell infiltration in pCR patients from the NIC and NAC cohorts was also significantly higher than non‐pCR patients (Figure 4C). It could be speculated that patients with high Treg cell infiltration before treatment might be easier to obtain pCR regardless of treatment modalities. Notably, the 7 differential signatures above did not differ significantly between pCR and non‐pCR patients in the NAC group. These findings further suggest that patients with relatively high expression of these 7 differential signatures are more prone to achieving pCR through NIC.

### 2.5 Correlation Between TIME Characteristics and DFS

**FIGURE 5:**
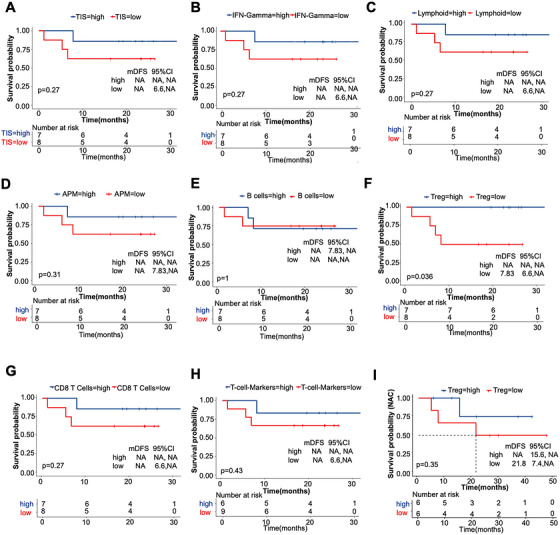
DFS of the patients stratified by the median score of the 7 signatures and Treg cell signature.

To further explore the correlation between TIME characteristics and DFS, we performed a correlation analysis between the 7 differential signatures specific to pCR patients in the NIC group and DFS. By dividing the patients into high‐ and low‐score groups according to the median score of the 7 signatures, we found that the high‐score group seemed to have better DFS than the low‐score group (Figure 5A–E,G–H), but no statistical difference was presented. In addition, based on the median score of the Treg cell signature, the patients were classified into high‐ and low‐score groups again. It was found that the patients with high Treg cell characteristics before treatment in the NIC group had significantly longer DFS than those with low Treg cell characteristics (*p* = 0.036, Figure 5F), and the same trend was also observed in the NAC group (Figure 5I).

In our previous study, we analyzed 11 DEGs between the pre‐pCR group and the pre‐non‐pCR group, as well as 148 genes related to TIME characteristics (whose differences were statistically significant). Among them, 7 genes were the intersection of the two groups. These 7 genes were used to establish a single‐gene prediction model for natural anti‐tumor efficacy (http://links.lww.com/JS9/B611). The final prediction model [neoadjuvant immunotherapy sensitivity score (NISS)] was constructed based on genes with a predictive ability greater than 0.9, that is, the average expression of TAP1 plus the expression of IRF4, which showed a significant difference between pre‐pCR and pre‐non‐pCR groups. Notably, the NISS model established in our previous study for predicting the response to NIC in TNBC [10] was used to validate its feasibility in the NAC cohort, and the results showed no significant differences between pCR and non‐pCR patients, with the area under the curve (AUC) of only 0.429 (Figure S1), further supporting the feasibility of the NISS model in predicting the response to NIC, but not NAC, in TNBC.

## 3 Discussion

In the present study, we demonstrated that addition of camrelizumab to NAC derived more clinical benefits in TNBC than the NAC alone, including increased pCR rates in both breast and axillae and decreased axillary dissection rates, while maintaining the similar BCS rates. Importantly, the patients with relatively high expression of the signatures like B cells, CD8 T cells, TIS, IFN gamma, lymphoid, APM, and T cell markers were more likely to achieve pCR through NIC.

Over the past few years, pCR has been confirmed as a prognostic factor for breast cancer [11]. Patients with pCR usually have better survival outcomes than those without pCR [12]. Considering the promising anti‐tumor activity of ICIs alone in a limited subset of patients, combined regimens of ICIs, such as chemoimmunotherapy, are increasingly applied in breast cancer. However, whether breast cancer patients undergoing NIC can achieve higher pCR rates than NAC alone remains ambiguous. In this study, the total pCR, breast pCR, and axillary pCR rates of patients treated with NIC were all significantly higher than those treated with NAC alone, suggesting that addition of camrelizumab to NAC could increase pCR rates in both breast and axillae, thus leading to the overall improved pCR in TNBC. This finding was consistent with previous studies [5, 10]. Anthracyclines have been demonstrated to exert direct cytotoxicity, enhance tumor immunogenicity and induce tumor cell death, and short‐term treatment with cisplatin and doxorubicin helps remodel a favorable TIME in TNBC to boost clinical responses to ICIs [13]. Nevertheless, antitumor immune response induced by ICIs may be damaged by steroid premedication for chemotherapy [14]. In view of this, nab‐paclitaxel, a drug without steroid premedication, and epirubicin were selected to increase the antitumor activity of camrelizumab. The total pCR rate of patients treated with NIC in our study was 64.8% whether before or after PSM, consistent with 64.8% reported in the study on neoadjuvant pembrolizumab plus platinum‐based chemotherapy [15] and slightly higher than 58.0% observed in the study on neoadjuvant atezolizumab plus sequential nab‐paclitaxel and anthracycline‐based chemotherapy [16]. In addition, other several trials on NIC showed relatively lower pCR rates [17, 18]. These variations in pCR rates may be partially attributed to different antitumor effects induced by different NIC regimens. In addition, subgroup analysis further indicated that the axillary pCR, breast pCR, and total pCR rates of node‐positive patients receiving NIC were all significantly higher than those receiving NAC, implying that neoadjuvant camrelizumab should be recommended for the patients with positive LNs. The suppressed activity of T cells in metastatic LNs is higher than that in the breast [19], which may be associated with less susceptible to the effect of NAC [20, 21, 22]. In node‐positive patients, systemic immune responses may be favorable for the response to ICIs [23], thus adding camrelizumab to NAC may enhance the axillary response in the LN‐involved patients. Further research is still needed to improve the patient selection for immunotherapy.

To date, the data regarding the prognostic benefit of preoperative immunotherapy remain scarce, especially for neoadjuvant camrelizumab in early‐stage TNBC. In this study, no significant differences in DFS were observed between the NIC and NAC cohorts, which might be associated with the short‑term follow‑up duration and limited sample size. Moreover, by assessing whether increased pCR rates translated into survival benefits, we observed that the patients with total pCR and breast pCR showed better DFS than those with residual tumors in the NIC cohort and the overall cohort, further highlighting the importance of pCR in translating the survival benefits based on the NIC. This finding was consistent with the previous studies suggesting that pCR was closely correlated with local control and favorable survival [20, 24].

By comparison to the pre‐treatment TIME characteristics of the pCR patients receiving NIC with those undergoing NAC, 7 unique signatures were identified in the NIC cohort, including B cells, CD8 T cells, TIS, IFN gamma, lymphoid, APM, and T cell markers, indicating that patients with these 7 differential signatures are more prone to obtaining pCR via NIC. Subsequently, through analysis of the correlation between TIME characteristics and DFS, we observed that the patients with high scores of the 7 signatures had the tendency of prolonged DFS compared with those with low scores. In a previous TNBC cohort, patients with increased B‐cell infiltration were found to have longer OS [25]. As the key targets of modulation by immune checkpoint inhibition, CD8^+ ^T cells with the tissue‐resident memory T (T_RM_) features was conductive to immunosurveillance in breast cancer [26], and a CD8^+^ T_RM_ gene signature was related to improved clinical outcomes in TNBC patients treated with ICIs [27]. In the pan‐cancer cohort, TIS score was identified to be correlated with complete or partial response to anti‐PD‐1 therapy, and patients with higher TIS scores showed prolonged OS than those with lower TIS scores [28]. In addition, as a classical antigen presentation process, APM was closely associated with different mutational patterns, immune cell infiltration, and expression of immunomodulators, and its‐related gene signature contributed to assessing the survival risk and guiding the ICI decision‐making for breast cancer patients [29].

Tumor microenvironment is infiltrated by various immune cells, in which Treg cells are frequently regarded as the negative regulators for immune responses. In our study, the proportion of Treg cell infiltration in pCR patients from both cohorts was significantly higher than non‐pCR patients before treatment, which further indicated that patients with high Treg cell infiltration might be easier to obtain pCR regardless of treatment modalities. Furthermore, the patients with high Treg cell characteristics from NIC and NAC cohorts revealed the tendency of better DFS than those with low Treg cell characteristics. These findings were supported by the previous study that FOXP3^+ ^tumor‐infiltrating lymphocytes were correlated with better pCR and OS [30].

Although PSM is used to minimize the confounding bias, this study still has some limitations. First, selection bias was inevitable due to the non‐randomized and hybrid prospective‐retrospective study design, which was inferior to rigorous two‐arm clinical trials or fully retrospective cohorts. Second, the relatively small sample size of the NIC cohort may compromise the robustness of our results. Third, the information regarding the number of patients ineligible for BCS prior to treatment was lacking, which may lead to overestimated BCS rates. In addition, the survival benefits could not be fully analyzed because of the short follow‐up duration. The relatively small sample size for TIME analysis including 16 samples in the NIC cohort and 12 samples in the NAC cohort may result in limited statistical power and affect generalizability of the observed findings. Future large‐scale prospective clinical trials with extended follow‐up are warranted to further validate our present findings.

In conclusion, neoadjuvant camrelizumab plus chemotherapy for TNBC contributes to improving pCR in both breast and axillae and decreasing axillary dissection, while maintaining the similar BCS rates. Patients with relatively high expression of 7 differential signatures are more likely to achieve pCR through NIC.

## 4 Materials and Methods

### 4.1 Study Design and Population

This was a prospective‐retrospective cohort study. The prospective cohort was from our previous open‐label, single‐arm, phase II trial (NCT04676997) on neoadjuvant camrelizumab plus chemotherapy for early‐stage TNBC [10]. Briefly, women aged ≥ 18 years with Eastern Cooperative Oncology Group (ECOG) performance status score of 0 or 1, and surgically resectable, stage II‐III (T1cN1‐2 or T2‐4N0‐2) TNBC were eligible. Patients with metastatic diseases, autoimmune diseases, history of concurrent malignancies, or history of severe allergic reactions to monoclonal antibodies, or immunosuppressive therapy were excluded.

Based on a previous phase II trial, 16 patients were enrolled into the NIC cohort in our current study, with trial enrollment completed on July 30, 2021. After trial closure, an additional 40 patients who received the same NIC regimen were retrospectively recruited and incorporated into the same NIC cohort. To further compare oncological outcomes after addition of immunotherapy, we retrospectively enrolled stage II‐III TNBC patients who only received conventional NAC at Breast Cancer Center, Shandong Cancer Hospital and Institute to form the NAC cohort. The inclusion and exclusion criteria of the NAC cohort were the same as those of the NIC cohort. Overall, the NIC cohort comprised 16 patients from the previous phase II trial and 40 additional patients treated with the same NIC regimen after trial completion. The retrospective NAC cohort included 220 patients. PSM was used to balance baseline characteristics between the two cohorts and reducing confounding bias.

The work has been reported in accordance with local regulations and Declaration of Helsinki. The study was approved by the Institutional Review Board of Shandong First Medical University and Shandong Academy of Medical Sciences Cancer Hospital (approval no.: SDZLEC2020‐022‐01), and written informed consent was obtained from all patients.

### 4.2 Treatment

As described in our previous study [10], patients in the NIC cohort received 4 cycles of camrelizumab (200 mg, d1, d15, q4w) in combination with nab‐paclitaxel (125 mg/m^2^, d1, d8, d15, q4w), and 4 cycles of camrelizumab (200 mg, d1, q2w) plus epirubicin (90 mg/m^2^, d1, q2w) and cyclophosphamide (600 mg/m^2^, d1, q2w). Patients in the NAC cohort were treated with 4 cycles of nab‐paclitaxel (125 mg/m^2^, d1, d8, d15, q4w), and 4 cycles of epirubicin (90 mg/m^2^, d1, q2w) plus cyclophosphamide (600 mg/m^2^, d1, q2w).

Surgery was usually performed with 2–4 weeks after the last cycle of neoadjuvant therapy, and surgical types were determined by the surgeon based on the radiological response. Sentinel lymph node biopsy (SLNB) was routinely performed. All patients received the dual tracer for sentinel lymph node (SLN) mapping, and at least 3 SLNs were detected. When lymph node metastasis in the neck was indicated on computerized tomography or ultrasonography, ALND was performed.

Postoperatively, the patients in both groups received camrelizumab once every 3 weeks for up to 9 cycles, regardless of the pathologic response. Of note, adjuvant camrelizumab plus capecitabine was recommended for patients with residual disease. Postoperative follow‐up was routinely conducted every 3 months during the first 2 years after surgery, then once every 6 months from 3 to 5 years.

### 4.3 Outcomes and Assessments

The primary endpoints included the total pCR, breast pCR, and axillary pCR rates. The secondary endpoint was DFS. The total pCR was defined as no invasive tumor cells in both the breast and axilla (ypT0/is ypN0). The LN response was defined as regression from histomorphologic evaluation or as downstaging based on the TNM classification. To accurately define the LN status, the regression of LNs was re‐evaluated according to the final histology, including negative LNs (no tumor involvement without evidence of regression), pCR (no metastasis with complete response in LNs), and non‐pCR (metastasis and no complete response in LNs). DFS was defined as the time from surgery to the date of disease progression, including locoregional recurrence and distant recurrence. Further TIME analysis of the gene expression was conducted on the samples obtained from the Phase II clinical trial, which constitutes an additional exploratory endpoint. The above research endpoints were compared between the NIC group and the NAC group, and were also re‐compared after PSM calculation.

### 4.4 Detection of RNA Expression

Nanostring nCounter PanCancer IO360 Panel (NanoString Technologies) was used to identify the gene expression profiling of 770 immune‐related genes comprising 20 housekeeping genes [31]. Formalin‐fixed paraffin‐embedded samples from the NIC cohort (*n* = 16; 15 samples before treatment and 10 samples after treatment) and the NAC cohort (*n* = 12; 12 samples before treatment and 11 samples after treatment) were collected, respectively, and the unstained slides of 5 µm thickness were obtained. According to the NanoString technote, RNA expression was detected. Normalized counts were log2 transformed finally for downstream analysis.

### 4.5 Analysis of DEGs and TIME Characteristics

DESeq2 R‐package was employed to identify the DEGs. The false discovery rate (FDR) was controlled to determine the *p*‐value threshold using Benjamini algorithm. The genes with FDR < 0.05 were defined as the DEGs. As described previously, the marker gene of TIME characteristics was retrieved [32]. In all immune cell types, the arithmetic means of genes were computed and used as the score for cell type indicators. The difference between the groups was analyzed using Wilcoxon test.

### 4.6 Statistical Analysis

Statistical analysis was performed using R software (version 3.6.1). Wilcoxon rank‐sum tests were used for comparison of continuous variables between groups, while Chi‐square or Fisher's exact tests were to compare the categorical variables between groups. PSM of the 2:1 ratio was performed to reduce the bias of confounding factors on the assessed outcomes between groups. Time‐to‐event curves for DFS were estimated using the Kaplan–Meier Method and compared by the log‐rank test. The value of *p* < 0.05 was considered statistically significant.

## Author Contributions

CHZ, YSW and PFQ participated in the study conception, design, and planning. YCW, XCM, JXZ, and PFQ were responsible for data acquisition. HQL, ZQS, and ZPZ participated in statistical analysis and interpretation of the results. XS, CJW and XJM provided technical and material support. CHZ drafted the manuscript, and YSW critically revised the manuscript. All authors reviewed the manuscript and agreed to submit it for publication.

## Funding

This work was supported by the Shandong Provincial Medical and Health Scienceand Technology Project (no. 202404080309);Shandong Provincial Natural Science Foundation (nos. ZR2026LSW057, ZR2024QH549); Weifang City Science and Technology Development Plan Project (no. 2024YX001); Wujieping Science Foundation of China (no. 320.6750.2024‐21‐73;no. 320.6750.2025‐21‐30); Science and Technology Development Project of the Affiliated Hospital of Shandong Second Medical University (no. 2024FYZ010).

## Ethics Statement

The study was approved by the Institutional Review Board of Shandong First Medical University and Shandong Academy of Medical Sciences Cancer Hospital (approval no.: SDZLEC2020‐022‐01) and by the Medical Research Ethics Committee of Weifang People's Hospital (approval no.: KYLL20251107‐9), written informed consent was obtained from all patients. Trial registration: ClinicalTrials.gov (NCT04676997)

## Conflicts of Interest

The authors declare that they have no competing interests.

## Supporting information



Supporting File: mco270924‐sup‐0001‐SuppMat.docx

## Data Availability

Raw data and analysis can be requested from the corresponding author via email with a reasonable request.
