# Supplementary material for: Clinical Efficacy of Neoadjuvant Camrelizumab Plus Chemotherapy for Triple‐Negative Breast Cancer and Tumor Immune Microenvironment Analysis: A Prospective‐Retrospective Cohort Study
Source: MedComm (2020). 2026 Aug 17;7(9):e70924. doi: 10.1002/mco2.70924 (PMC13478840; doi:10.1002/mco2.70924)
Supplement: Supplementary file 1 [file MCO2-7-e70924-s001.docx]

Title page:

Title: Clinical efficacy of neoadjuvant camrelizumab plus chemotherapy for triple-negative breast cancer and tumor immune microenvironment analysis: A prospective-retrospective cohort study

Authors: Yongsheng Wang1, Pengfei Qiu1# Yuechen Wang2，Xingchen Meng2, Jiaxin Zhang2, Haiqiang Liu3, Zhiqiang Shi1, Zhaopeng Zhang1, Xiao Sun1, Chunjian Wang1, Xiangjing Meng4, Chunhui Zheng2*

Main Authors：Yongsheng Wang1, Pengfei Qiu1#

Main author’s affiliation: Breast Cancer Center, Shandong Cancer Hospital and Institute, Shandong First Medical University and Shandong Academy of Medical Sciences, Jinan, China

Corresponding author:

Chunhui Zheng, M.D, Breast Disease Center, Weifang People's Hospital, Shandong Second Medical University, Weifang, Shandong Province, China., No. 151 Guangwen Road, Weifang, China. Tel.: 0536-8192208; E-mail address: [rmyychunhuizheng@sdsmu.edu.cn](mailto:rmyychunhuizheng@sdsmu.edu.cn).

Short Title: Neoadjuvant camrelizumab plus chemotherapy for early TNBC

Figure S1.

**Figure S1** Comparison of the NISS between pCR and non-pCR patients and the receiver operator characteristic curve of the NISS in Shandong TNBC cohort (A-B); Comparison of the NISS between pCR and non-pCR patients and the receiver operator characteristic curve of the NISS in I-SPY2 TNBC cohort (C-D); Comparison of the NISS between pCR and non-pCR patients and the receiver operator characteristic curve of the NISS in TNBC cohort treated with NAC (E-F).
